# eDNA Analysis of Aquatic Microcosms Reveals the Effects of a Copper Pollution Event on Sedimentary Microbial Assemblages in Freshwater Wetlands

**DOI:** 10.1002/ece3.74180

**Published:** 2026-08-11

**Authors:** Anna S. Flynn, A. Mark Osborn, Vincent Pettigrove, Jeff Shimeta, Sara M. Long

**Affiliations:** ^1^ Aquatic Environmental Stress Research Group RMIT University Bundoora Victoria Australia; ^2^ School of Science RMIT University Melbourne Victoria Australia

**Keywords:** bacteria, biomonitoring, environmental DNA, metabarcoding, metals, wetlands

## Abstract

Metal contamination is known to cause shifts in structure of freshwater assemblages where sensitive species do not survive and tolerant species thrive, and given their sensitivity, ubiquitous nature, and high abundances, microbes are ideal targets for biomonitoring assessments. However, the responses of diverse microbial communities to metal contamination in freshwater environments are currently understudied, particularly in the Southern hemisphere. Therefore, in this study we used environmental DNA metabarcoding to assess the influence of copper contamination events on an urban wetland. Laboratory‐based microcosms with clean and contaminated sediments were spiked with copper sulphate to assess microbial responses. We saw significant microbial variation both over time and between concentrations, with responses varying between the microbes from uncontaminated and contaminated sediments; outlining how the dynamics develop over a concentration and temporal gradient and how they vary with assemblages from contaminated and uncontaminated environments, with seven families identified as possible indicators of metal contamination.

## Graphical abstract

This study used eDNA metabarcoding to assess the impacts of copper contamination on freshwater wetlands, finding that microbial community structure varies significantly with metal concentration, over a temporal scale, and between microbial communities from unpolluted and polluted wetlands. We highlight seven bacterial families as possible indicators of metal contamination.

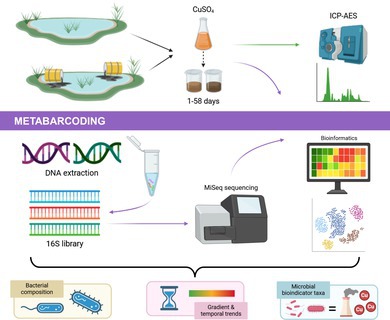

## 1 Introduction

Globally, freshwater systems are experiencing rapid declines in biodiversity and overall health (Higgins et al. 2021; Čehovská et al. 2022), partly due to expanding urbanisation increasing the volume of anthropogenic contaminants entering waterbodies (Dias‐Ferreira et al. 2016). Constructed wetlands (CWs) play an important role in protecting and improving water quality to preserve downstream waterbodies through the retention of pollutants, being increasingly utilised in water sensitive urban design (Zhu et al. 2019). CWs are generally expected to contain a variety of contaminants, like metals, which are representative of the surrounding land use and most prevalent anthropogenic contaminants. The majority of contaminants in wetlands are found within sediments which store the contaminants that have been removed from the water (Pawlowski et al. 2022).

Sedimentary microbial assemblages are frequently exposed to contaminants entering CWs, which can impact ecological functioning directly as well as eliciting more complex indirect responses in microbial assemblage structure and function (Yang et al. 2018). Shifting taxonomic diversity and composition in an ecosystem is known to alter ecological functioning even without a reduction in species richness (Spaak et al. 2017), impacting processes such as biochemical functioning and primary production (Yang et al. 2018). Heavy metals in particular pose a significant threat to freshwaters and microbial assemblages due to their persistence in the environment, high toxicity, and high rates of bioaccumulation (Zhang et al. 2016). Copper (Cu) is a widespread contaminant of concern (Yang et al. 2018) and is acutely toxic to aquatic biota even in trace amounts (Yang et al. 2018; ANZG 2018). Copper strongly adsorbs to suspended particles and sediments (ANZG 2018), often decreasing microbial phylogenetic diversity (Mahamoud Ahmed et al. 2020).

Environmental DNA (eDNA) analysis has a wide scope of applications including evaluating the correlations between taxonomic diversity and composition and the chemical profile of an ecosystem (Pawlowski et al. 2021). While the use of DNA assessments to identify microbial communities is not new, the introduction of microbial ecology tools into standard biomonitoring practices via eDNA studies is a relatively recent endeavour (Pawlowski et al. 2021). Significant gaps still exist that restrict the implementation of microbial eDNA analysis as a biomonitoring tool for freshwater ecosystems, including how microbes respond to chemical mixtures, how responses vary between assemblages from differing background environments, and how the responses vary through taxonomic ranks from phylum down to species level. Studies assessing the utilisation of microbes as bioindicators in freshwater are somewhat limited, with those that do exist focusing mainly on the impact of heavy metals received directly from nearby anthropogenic activities (Li et al. 2020; Custodio et al. 2022; Staebe et al. 2018). A notable geographical imbalance also exists, with a significant majority of eDNA studies being performed in the Northern hemisphere (Schenekar 2023; Takahashi et al. 2023).

In this study, laboratory‐based microcosms spiked with Cu were analysed by eDNA metabarcoding to evaluate the influence of Cu contamination on the composition of wetland sedimentary microbial assemblages from an uncontaminated wetland and a contaminated wetland. The main aim of this study was to assess the impact of a Cu contamination event on sedimentary microbial assemblages, to determine if previous pollution exposure influences microbial sensitivity, and to identify potential taxa that could be used as bioindicators for metal pollution. We hypothesised that a larger spike would result in a stronger microbial response, that the microbial responses will develop over time and may stabilise by the conclusion of the experiment, and that the microbes originating from an environment with previous pollution will be less sensitive (show less response) to subsequent pollution events. This study was designed to reproduce the conditions which would be experienced as a result of a copper pollution event in a CW, natural wetland, or otherwise shallow waterbody, with the combined effects of both copper toxicity and physicochemical implications from the metal input.

## 2 Methods

### 2.1 Experimental Design

This experiment consisted of two sediment types, spiked with one of three Cu concentrations, and sampled at five time points. Each treatment‐time point combination consisted of three replicate microcosms, totalling 90 individual samples. In an effort to maintain an intact aerobic layer of sediment and not disturb the wider system with each sample collection, each microcosm was sampled only once, at its designated time point. Each microcosm was a 250 mL glass beaker, covered with a thin mesh to allow for adequate air exchange. The three treatments consisted of a control at 0 mg/kg (Cu/sediment), a concentration at the Australian and New Zealand freshwater toxicity guideline (ANZG) default guideline value (DGV) of 65 mg/kg, and a concentration at the ANZG high guideline value (HGV) of 270 mg/kg (ANZG 2018). These concentrations were achieved by spiking the microcosms with one quarter of the final Cu concentrations, delivered in solution with water as the solvent, once per week over a four‐week period. The spiking regime used in this experiment was determined from a pilot study. The quarter dose spikes were 0, 16.25, and 67.50 mg/kg for the control, DGV, and HGV treatments, respectively. Samples were collected on days 0, 2, 11, 28, and 56, while spiking occurred on days 1, 8, 15, and 22. This experiment was run during late winter to early spring, from September 1st through to October 31st, 2023.

### 2.2 Microcosm Preparation

Surficial sediments (top 2 cm) were collected from two wetlands, one contaminated urban stormwater wetland (GLE) and one uncontaminated rural wetland (BIT), and filtered through a 63 μm net. Three replicate samples of each sediment type were used to calculate sediment moisture content by drying for 24 h in a 60°C oven and using the formula described in Equation S1, Appendix A.

Each beaker was pre‐weighed, filled with 50 g of wet sediment, or roughly 1–2 cm depth of sediment, then re‐weighed. The beaker was filled with 20 mL of chironomid water, gently added by syringe onto the sediment surface to avoid disturbing it. Chironomid water is an artificial water prepared from a modified version of Martin's solution (RO water with 0.12 mM NaHCO_3_, 0.068 mM CaCl_2_, 0.083 mM MgSO_4_, 0.86 mM NaCl, 0.015 mM KH_2_PO_4_, 0.089 mM MgCl_2_, and 0.1% (w/v) iron) (Martin et al. 1980). Once filled with sediment and chironomid water, the beakers were covered with a layer of cheesecloth, secured by a rubber band. The microcosms were placed inside a greenhouse outdoors, positioned along a shaded fence. Three biological replicates were used per treatment.

### 2.3 Spiking, Sampling, and Maintenance

The microcosms were spiked with a stock solution of CuSO_4_ at 2.5 g Cu/L concentration or chironomid water (for the controls). The exact spiking volume for each individual microcosm was calculated from the beaker weights, sediment weights, and sediment moisture content using the formula described in Equation S2, Appendix A. The DGV and HGV microcosms were spiked with the Cu solution (averaging 200 μL for each DGV spike and 500 μL for each HGV spike) and the controls were spiked with 350 μL of chironomid water.

Each microcosm beaker was sacrificed upon sampling. The cheesecloth was removed from each microcosm, and the surface water was transferred by syringe into a new, clean beaker for temperature and pH measurements. The water was then decanted into a 50 mL syringe and filtered through a 0.22 μm filter into a plastic bottle and preserved with nitric acid. The top aerobic layer of sediment was scraped off the top of each microcosm, deposited into a labelled 2 mL Eppendorf tube, and stored at −80°C. The remaining sediment was collected and stored at 4°C until metal analysis.

Over the course of the 8‐week experiment, the microcosms were checked regularly to ensure water levels remained around the initial fill level. When the water levels dropped due to evaporation, they were replenished with chironomid water to the original fill line.

### 2.4 Chemical and Microbial Analysis

For chemical analysis, both sediment and water samples were sent to Australian Laboratory Services (ALS) (Springvale, Australia). For each sediment, treatment, and time point, one replicate pooled sample was analysed. Each sample was analysed for concentrations of Cu, Cr, As, Ni, Pb, Zn, and Cd by ICP‐AES. For microbial analysis, DNA was extracted from 250 mg of sediment from each sample with a DNeasy PowerSoil Pro extraction kit (Qiagen, Clayton, Australia) using the spin‐column protocol, along with extraction blank controls (DNase/RNase‐Free Distilled Water, Thermo Fisher, Scoresby, Australia). The samples were analysed by NanoDrop microvolume spectrophotometer (Thermo Fisher, Scoresby, Australia) to assess purity, quantify DNA extracts, and to ensure no DNA contamination in the controls. The samples were diluted to 5–10 ng DNA/μL and 20 μL pipetted into a 96‐well plate, one sample per well, arranged in columns (leaving two empty cells for positive and negative PCR controls), and stored at −80°C before being sent to Ramaciotti Centre for Genomics (UNSW, Sydney, Australia) for 16S v3‐v4 amplicon library preparation and 2 × 300 bp paired‐end Illumina MiSeq sequencing. The library preparation and sequencing analysis were completed according to the manufacturer's protocols (Illumina 2013), as described in a previous study (Flynn et al. 2025), with amplicon PCR (95°C for 3 min; 25 cycles of: 95°C for 30 s, 55°C for 30 s, 72°C for 30 s; 72°C for 5 min; Hold at 4°C) using Illumina overhang primers and locus‐specific primers (16S_341f, 16S_805r); amplicon cleaning using AMPure XP beads (Beckman Coulter, Mount Waverly, Australia); indexing PCR with Nextera compatible IDT10 unique dual indexes (N7XX, S5XX); sample normalisation using SequalPrep kit (Thermo Fisher, Scoresby, Australia); sample elution in 20 ul buffer with equal volumes used for pooling; and pooled sample cleaning using AMPure XP beads (Beckman Coulter, Mount Waverly, Australia). Quantification and molarity calculations were determined by TapeStation (Agilent, Santa Clara, USA) and Qubit (Thermo Fisher, Scoresby, Australia).

Bioinformatic analysis of the microbial sequences was performed using the QIIME 2 (v 2022.8) platform (Bolyen et al. 2019; Bai et al. 2019), run through the RMIT University Amazon Web Services (AWS) cloud supercomputing hub (RACE). Primers were removed from the sequences using the q2‐cutadapt plugin (Martin 2011) before filtering, trimming, and denoising into amplicon sequencing variants (ASVs) (Callahan et al. 2016) using the trim parameters trim‐left 5 for forward and reverse sequences and trunc‐len 240 for forward and trunc‐len 220 for reverse sequences. Chimeric sequences were removed following the DADA2 (v. 1.16.0) pipeline (Callahan et al. 2016), and samples with < 10,000 reads removed. Taxonomy was assigned using the Greengenes2 (v 2022.10) classifier (McDonald et al. 2023) and a phylogenetic tree constructed from masked alignments using the q2‐phylogeny plugin. Diversity analyses were calculated using the q2‐diversity plugin on data rarefied to a sampling depth of 20,000. Classified ASV relative frequency tables were exported out of QIIME and into PRIMER‐e (v7) for multivariate analyses including non‐metric multidimensional scaling (nMDS) plot, analysis of similarities (ANOSIM), SIMPER analysis (Clarke 1993), and BEST analysis (Clarke et al. 2014). The sequence data were log (x + 1) transformed and a Bray–Curtis similarity matrix constructed, which was used in nMDS, ANOSIM, and BEST analyses, while the SIMPER analysis was conducted on ASV relative frequency tables. The nMDS ordination was used to visually assess sample groupings, with ANOSIM providing statistical analysis of those sample groupings, to determine if treatment or temporal factors are causing significant changes to microbial community structure. Two‐way SIMPER analysis provides information on which bacterial taxa are contributing to any significant sample group differences found from the above analyses, while BEST analysis correlates environmental factors which may be causing the bacterial taxa variability between sample groups, to determine what environmental contaminants have the biggest impact on microbial structures and which microbes are sensitive or tolerant. A significance level of α ≤ 0.05 was used in all analyses. For the ANOSIM analysis, an R statistic value of ≥ 0.75 is considered well separated (Clarke 1993), so was used as the threshold to determine significant difference between samples. Chemical variables are compared to ANZG freshwater toxicity guideline values (ANZG 2018).

A toxic unit (TU) quotient was used to assess the total toxicity of the water from the sum of the extents by which each metal exceeded its trigger value for days 28 and 56 after spiking concluded. The TU calculation is described in Equation S3, Appendix A. Results for chemicals that had no concentrations which exceeded the trigger value were not reported. TU provides a measure of total toxicity for each treatment as well as each metal.

## 3 Results

### 3.1 Cu Concentration in Treatments

**FIGURE 1:**
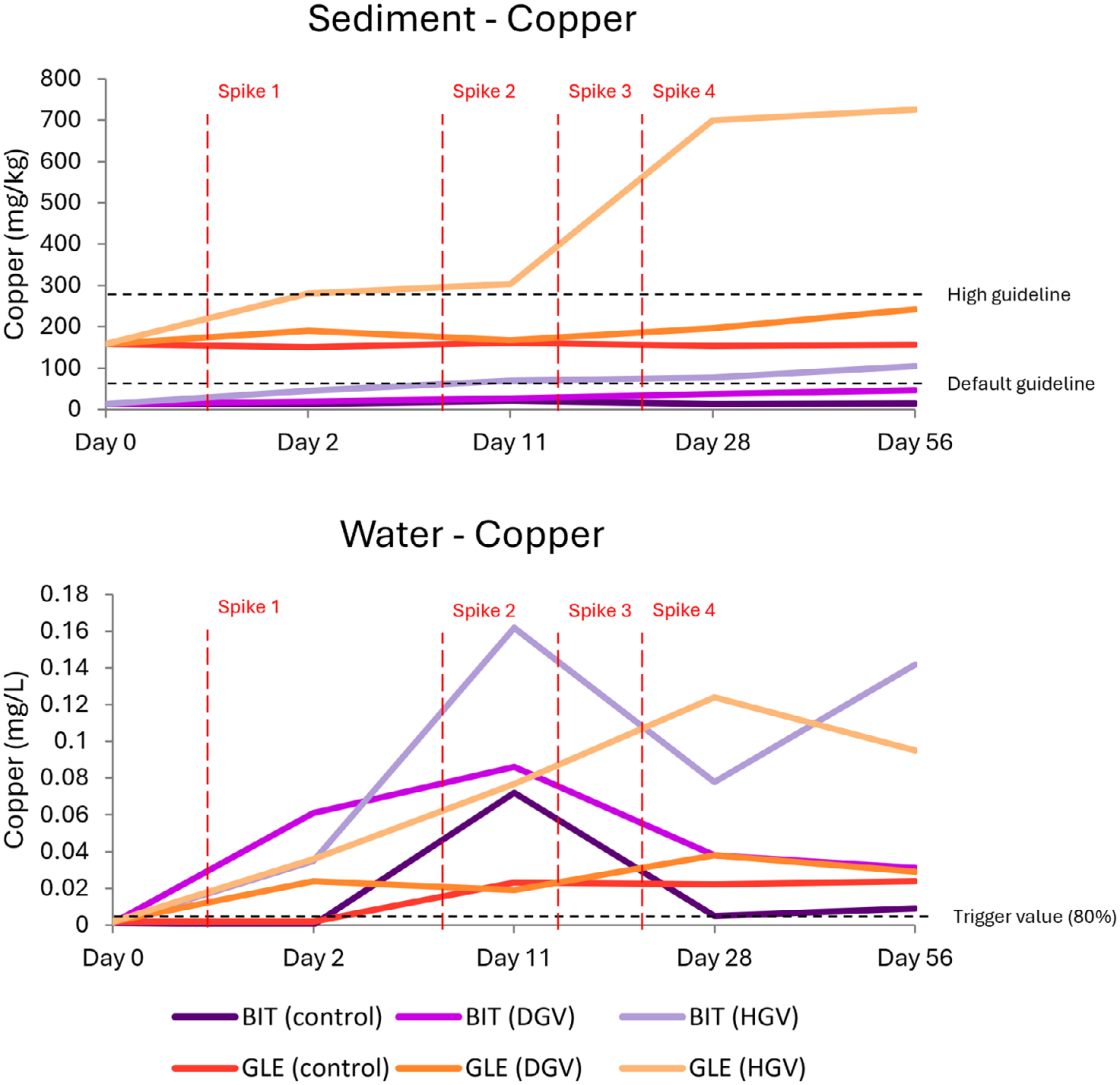
Copper concentrations measured from BIT and GLE sediment (top) and water (bottom) samples at five time points (0, 2, 11, 28, 56) for three treatments (control, DGV, HGV). The dotted lines represent the ANZG (ANZG 2018) default and high guideline values for sediment and the 80% species protection trigger value for water. The red dotted lines indicate when spiking occurred.

Over the course of the experiment, Cu concentrations increased in all the spiked treatments in both sediments, while the control treatments remained relatively stable with only a slight increase in Cu concentrations on day 11 (Figure 1). Cu concentrations in the BIT samples ranged from 13 mg/kg on day 0 to 104 mg/kg in the HGV day 56 sample. The HGV BIT samples were above the default guideline value from day 11 onwards, while all other BIT samples were below guideline values. Cu concentrations in the GLE samples ranged from 150 mg/kg in the control day 2 sample to 726 mg/kg in the HGV day 56 sample, increasing sharply between day 11 and day 28 (Figure 1). The HGV GLE samples were above the high guideline value from day 2 onwards, and all other GLE samples were above the default guideline value.

Cu concentrations in the water samples fluctuated significantly more than in the sediment samples, following no discernible pattern over time or treatment (Figure 1). Concentrations ranged from below detection level (< 0.001 mg/L) to 0.162 mg/L for BIT and from 0.02 mg/L to 0.124 mg/L for GLE. Overall, Cu concentrations were higher in the BIT samples than in the GLE samples, with both sediments experiencing peaks on day 11. Cu was below detection level in one sample (BIT control day 2), below the 80% species protection trigger value (0.0025 mg/L) in all day 0 samples and the GLE control day 2 sample, and above the trigger value in all other samples (Figure 1). Cu was the biggest contributor to the TU quotient across all samples. It contributed 5.60, 27.6, and 88.0 TU to the BIT control, DGV, and HGV samples, respectively, and 18.4, 26.8, and 87.6 TU to the GLE control, DGV, and HGV samples, respectively (Figure 3).

### 3.2 Concentrations of Metals in Uncontaminated and Contaminated Sediment Samples

All sediment samples showed a similar trend of high metal concentrations in the GLE samples and low metal concentrations in the BIT samples, regardless of treatment (Figure S1, Appendix D). Detection limits and ANZG (ANZG 2018) 80% trigger values for all metals are listed in Table S1, Appendix B. Arsenic (As) was below detection level in all BIT samples and below guideline levels in all GLE samples, averaging 13 mg/kg across the sample period. Nickel (Ni) concentrations averaged 3 mg/kg for BIT, which is below guideline values, and 45 mg/kg for GLE, which exceeds the default sediment guideline value. Chromium (Cr) concentrations averaged 8 mg/kg in the BIT samples, below guideline values, and 110 mg/kg in the GLE samples, above the default sediment guideline value. Zinc (Zn) concentrations averaged 7 mg/kg for BIT, below guidelines, and 1718 mg/kg for GLE, which exceeds the high guideline value. Cadmium (Cd) concentrations were below detection level for BIT and averaged 2 mg/kg for the GLE samples, exceeding the default guideline value. Lead (Pb) concentrations averaged 7 mg/kg for BIT, below all sediment guideline values, and 56 mg/kg for GLE, which was above the default sediment guideline value (Figure S1, Appendix D).

**FIGURE 2:**
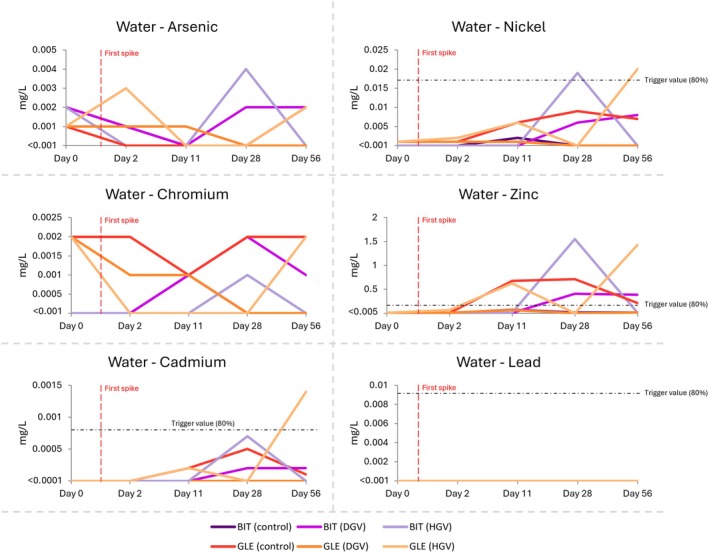
Metal concentrations measured from BIT and GLE water samples at five time points (0, 2, 11, 28, 56) for three treatments (control, DGV, HGV). A black dotted line represents the ANZG (ANZG 2018) 80% species protection trigger value for water. A red dotted line indicates when the initial spiking occurred.

**FIGURE 3:**
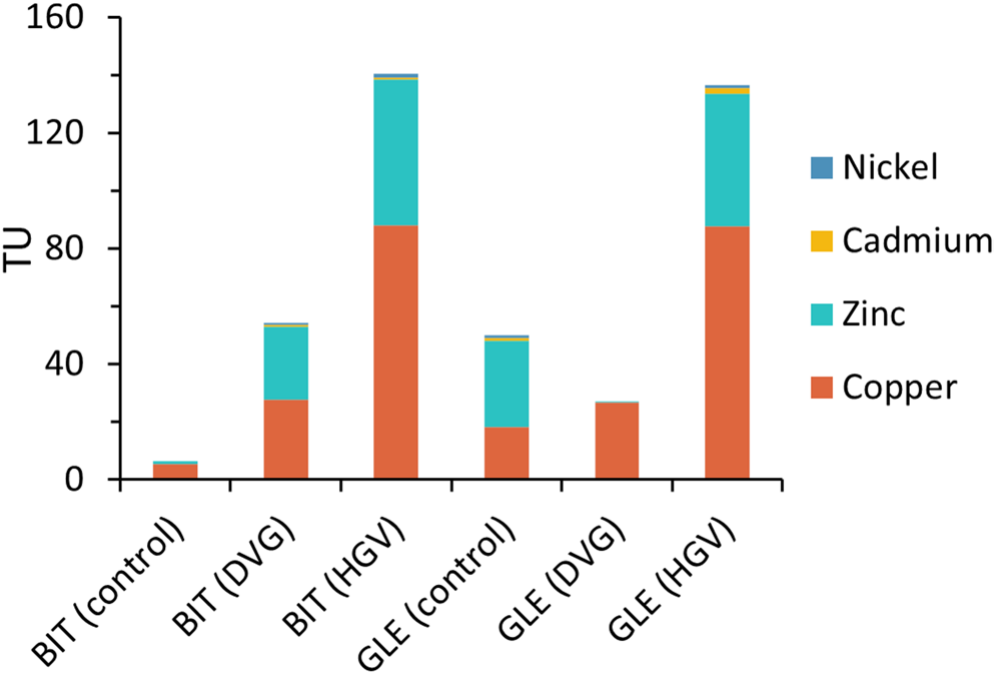
Stacked bar graph of the contribution of each metal to the total TU values from BIT and GLE water samples for three treatments (control, DGV, HGV) combined from two time points (28, 56). Only metals which exceeded the 80% species protection trigger value (ANZG 2018) are shown.

Metal concentrations in the water samples were considerably more variable than in the sediment samples, although the fluctuations were very minor in terms of actual concentration (Figure 2). Detection limits and ANZG (ANZG 2018) DGV & HGV trigger values for all metals are listed in Table S2, Appendix B. Arsenic concentrations were below detection levels in three BIT samples and three GLE samples, otherwise peaking at 0.004 mg/L which does not exceed the 80% species protection trigger value. Nickel was below detection levels in six BIT samples. All other samples ranged up to 0.02 mg/L, exceeding the trigger value on two occasions (Figure 2). Nickel contributed 0, 0.824, and 1.12 TU to the BIT control, DGV, and HGV samples, respectively, and 0.941, 0, and 1.18 TU to the GLE control, DGV, and HGV samples, respectively (Figure 3). Chromium concentrations ranged from below detection level to 0.002 mg/L among all samples, not exceeding the trigger value in any samples. Zinc concentrations were above detection levels in all but four samples. All other samples ranged between 0.005 to 1.55 mg/L, exceeding the trigger values in one third of those (Figure 2). Zinc was the second biggest contributor to the TU quotient across all samples. It contributed 0.903, 25.5, and 50.4 TU to the BIT control, DGV, and HGV samples, respectively, and 29.8, 0.452, and 46.1 TU to the GLE control, DGV, and HGV samples, respectively (Figure 3). Cadmium concentrations were below detection levels in all samples up to day 11, then peaked at 0.0014 mg/L exceeding the trigger value in that sample (Figure 2). Cadmium contributed 0, 0.500, and 0.875 TU to the BIT control, DGV, and HGV samples, respectively, and 0.750, 0, and 1.75 TU to the GLE control, DGV, and HGV samples, respectively (Figure 3). Lead concentrations were below detection limits in all samples (Figure 2). In total, the BIT samples were exposed to 6.50, 54.4, and 140 TU for the control, DGV, and HGV samples, respectively, and the GLE samples were exposed to 59.8, 27.3, and 136 TU for the control, DGV, and HGV samples, respectively. Overall, the metals can be divided into two groups: high impact (Cu, Zn) and low impact (Cd, Cr, Ni), based on their contributions to the final TU quotients, and the samples can be categorised into three groups: high toxicity (BIT HGV, GLE HGV), medium toxicity (BIT DGV, GLE control, GLE DGV), and low toxicity (BIT control), based on their final TU quotient (Figure 3).

### 3.3 Relationship Between pH and Copper Concentrations

**FIGURE 4:**
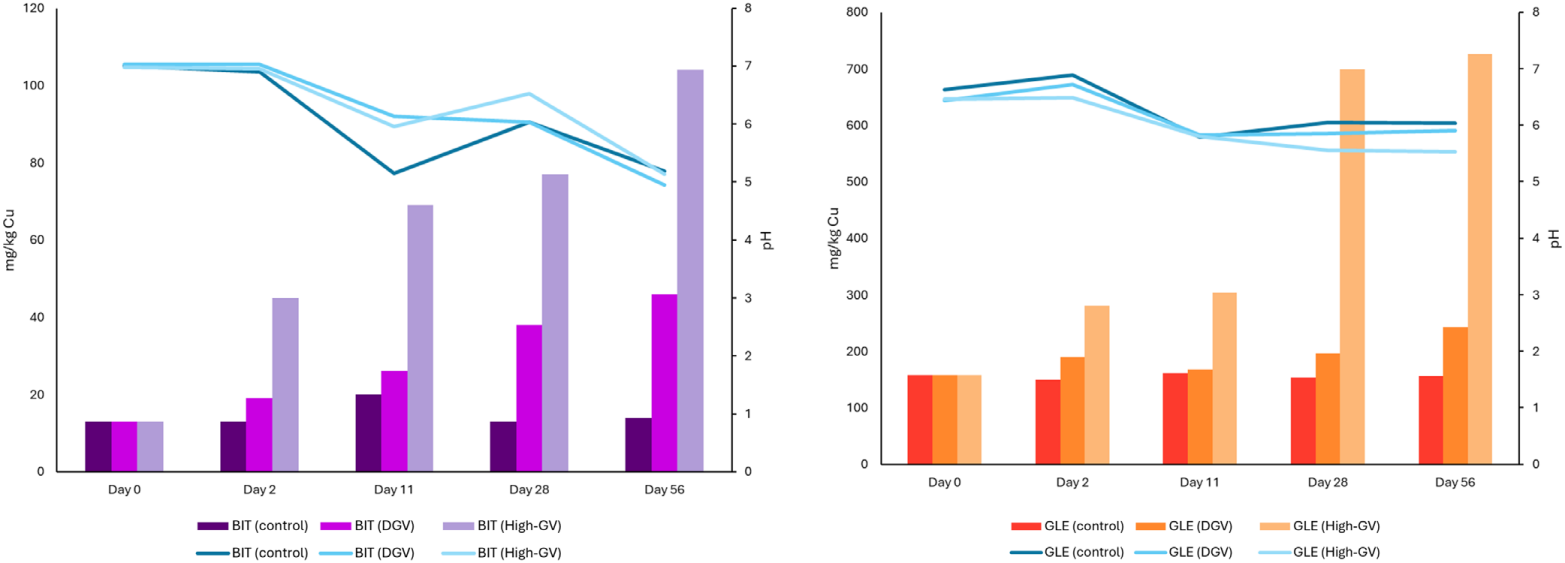
Comparison of copper concentrations (bars, primary axis) and pH (lines, secondary axis) for BIT (left) and GLE (right) sediments.

The addition of copper, via CuSO_4_, into the microcosms saw a consequent decrease in pH. In the BIT samples, pH decreased from ~7 at day 0 of the experiment to ~5 at day 56 across all treatments. Similarly, in the GLE samples, pH sat at ~6.5 at day 0 of the experiment before dropping to ~6 at day 56 for all treatments with the exception of the HGV treatment which dropped to 5.5 pH. While Cu concentrations rose steadily, pH fluctuated with an overall decrease, which was comparable between all treatments (Figure 4).

### 3.4 Next‐Generation Sequencing (NGS) Overview

The Illumina MiSeq sequencing run generated a total of 14,609,985 paired‐end raw reads from 90 microcosm sediment DNA samples. After quality control, denoising, and removal of potential chimeric sequences, 7,435,453 sequences were retained. The average number of eligible reads per sample was 84,493 (min: 12,314, max: 176,764). After filtering and rarefaction, 88 samples were retained. From the high‐quality reads, a total of 99 phyla, 263 classes, 712 orders, 1355 families, 2583 genera, and 3556 species were classified.

### 3.5 Microbial Assemblage Diversity

**FIGURE 5:**
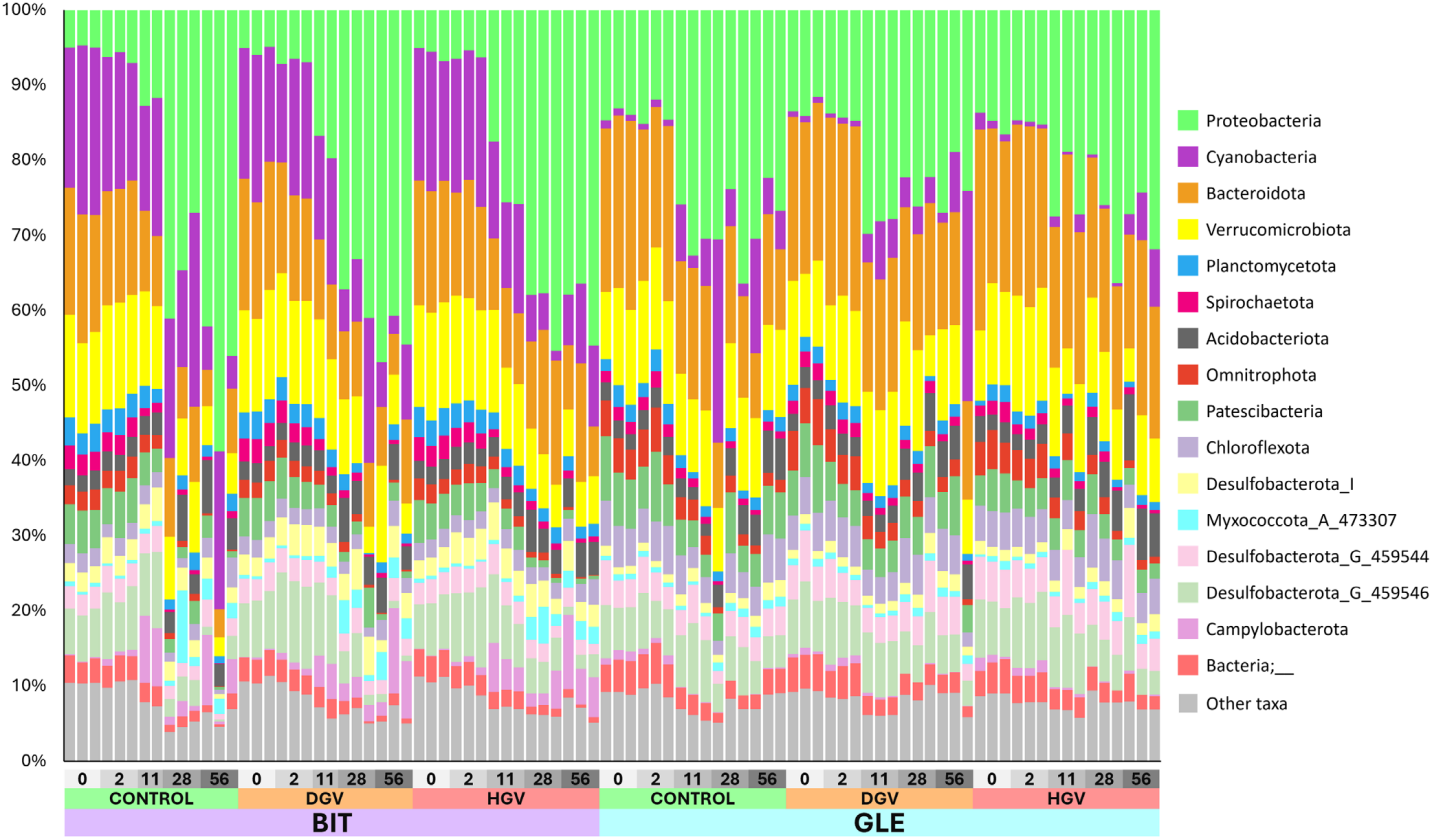
Relative frequency bar plot of all samples from 16S DNA, shown at phylum level. Only dominant taxa (> 1% overall abundance) are displayed separately, all other taxa are combined into ‘other taxa’. Samples shown left to right are day 0, 2, 11, 28 & 56 for each treatment (control, DGV, HGV) for BIT and GLE, respectively.

The bulk of the high‐quality reads (7,397,439; 99.5%) were classified as Bacteria, while the remaining reads were classified as either Archaea (34,852; 0.46%) or were unassigned (3162; 0.04%). Of the 99 identified phyla, 14 were considered dominant (> 1% of total abundance). Proteobacteria was the most dominant phylum accounting for 22% of total abundance, followed by Bacteroidota with 16% of total abundance. Desulfobacterota, Verrucomicrobiota, and Cyanobacteria accounted for 12%, 11%, and 9% of total abundance, respectively. The next most abundant phylum was Patescibacteria with 4% of total abundance. Chloroflexota, Acidobacteriota, Omnitrophota, Planctomycetota, and ASVs unassigned at phylum level each accounted for 2%–4% of total abundance, while Campylobacterota, Spirochaetota, and Myxococcota each accounted for < 2% of total abundance. A temporal trend of increasing Proteobacteria abundance over time was present across all samples but was more pronounced in the BIT samples than the GLE samples. The relative abundances of Bacteroidota and Verrucomicrobiota were relatively similar between the BIT and GLE samples; Cyanobacteria, however, was much more abundant in the BIT samples (Figure 5).

### 3.6 Temporal Distribution of Microbial Assemblages

**FIGURE 6:**
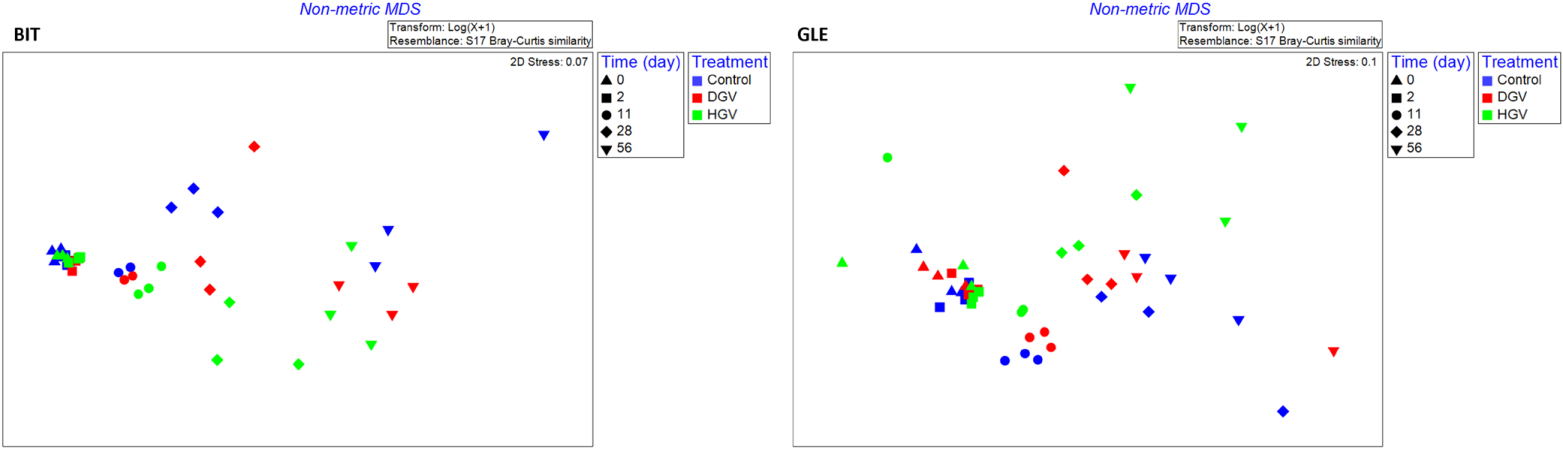
nMDS ordination plot of samples from the two sediment types, BIT (left) and GLE (right), determined from 16S rRNA gene sequencing with sequences identified at ASV level. Samples are split by time (day 0, 2, 11, 28, 56) and treatment (control, DGV, HGV).

Comparing between treatments based on identification of taxa at ASV level, an nMDS ordination plot showed a slight visible separation between treatment groups, moving from the control through to DGV followed by HGV (Figure 6). Pairwise ANOSIM analysis confirmed there was a significant difference between treatment groups for BIT (global *R* = 0.35, *p* = 0.001, 999 permutations) and GLE (global *R* = 0.151, *p* = 0.006, 999 permutations), but low separation. In the BIT samples, the control was significantly different from both spiked treatments, but for GLE the control was only significantly different from the HGV, although the two spiked treatments were significantly different from each other. Although none of the pairwise R values exceed the *R* ≥ 0.75 benchmark for high separation, there are clear trends across both sediments where the control/HGV has the highest R statistic. The higher R value for control/HGV compared to control/DGV highlights the influence of copper concentration, not just a presence/absence effect (Table 1). Pairwise ANOSIM also confirmed the significant difference between time points, with all BIT pairs (global *R* = 0.768, *p* = 0.001, 999 permutations) and all GLE pairs (global R = 0.711, p = 0.001, 999 permutations) being significantly different from each other. Only one BIT pair (11/28) and three GLE pairs (0/2, 11/28, 28/56) were not highly separated, with R values lower than 0.75 (Table 2). An nMDS ordination plot at phylum level further highlights the significant change in microbial assemblage over time (Figure S2, Appendix E). Overall, the BIT samples appear to have more spread compared to the GLE samples. For both sediment types, the spread of the data increased over time, with the earlier time points being less variable compared to the later time points. Similarly, the two sediment types become closer over time, moving from two distinct groups at day 0 to having overlapping data points by day 56 (Figure S2, Appendix E).

**TABLE 1:** Pairwise ANOSIM results comparing each treatment (control, DGV. HGV) for BIT and GLE respectively, determined from 16S DNA. Significant results (*p* < 0.05, *R* > 0.75) are italicised.

Groups (treatment)	R statistic	*p*
BIT		
Control, DGV	0.388	*0.003*
Control, HGV	0.591	*0.001*
DGV, HGV	0.103	0.157
GLE		
Control, DGV	0.03	0.37
Control, HGV	0.267	*0.003*
DGV, HGV	0.237	*0.004*

**TABLE 2:** Pairwise ANOSIM results comparing each time point (day 0, 2, 11, 28, 56) for BIT and GLE respectively, determined from 16S DNA. Significant results (*p* < 0.05, *R* > 0.75) are italicised.

Groups (day)	R statistic	*p*
BIT		
0, 2	*0.864*	*0.002*
0, 11	*1*	*0.003*
0, 28	*0.963*	*0.002*
0, 56	*1*	*0.001*
2, 11	*1*	*0.001*
2, 28	*0.926*	*0.001*
2, 56	*1*	*0.003*
11, 28	0.741	*0.007*
11, 56	*1*	*0.005*
28, 56	*0.988*	*0.002*
GLE		
0, 2	0.259	*0.021*
0, 11	*0.765*	*0.003*
0, 28	*0.84*	*0.002*
0, 56	*0.889*	*0.001*
2, 11	*0.84*	*0.001*
2, 28	*0.889*	*0.003*
2, 56	*0.889*	*0.002*
11, 28	0.531	*0.001*
11, 56	*0.79*	*0.001*
28, 56	0.185	*0.019*

### 3.7 Relationship Between Microbial Structure and Chemical Occurrences

**FIGURE 7:**
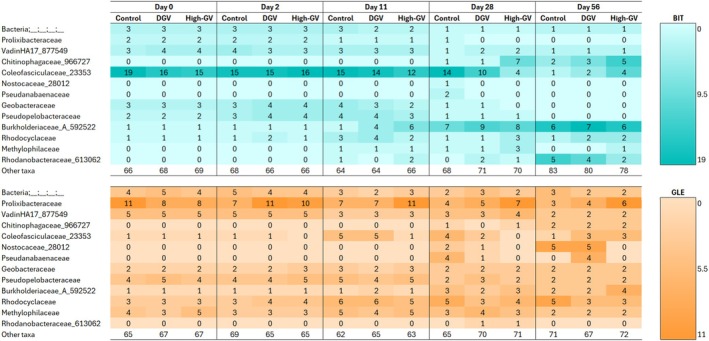
Heatmap showing the relative abundances (%) of 13 families from the two sediment type samples, BIT (blue) and GLE (orange), determined by SIMPER analysis from 16S DNA.

Thirteen families were identified through two‐way SIMPER analysis as contributing most to the microbial variability seen between samples, including ASVs unassigned at phylum level, shown through their changing relative abundances and contribution to community variability. Comparing sediment origins, two Bacteroidota families (Prolixibacteraceae, VadinHA17_877549), two Cyanobacteria families (Nostocaceae_28012, Pseudanabaenaceae), one Desulfobacterota family (Pseudopelobacteraceae), and two Proteobacteria families (Rhodocyclaceae, Methylophilaceae) had higher abundances overall in the more polluted GLE sediment. Contrarily, one Bacteroidota family (Chitinophagaceae_966727), one Cyanobacteria family (Coleofasciculaceae_23353), and two Proteobacteria families (Burkholderiaceae_A_592522, Rhodanobacteraceae_613062) had higher abundances overall in the less polluted BIT sediment. The remaining Desulfobacterota family (Geobacteraceae) showed no noticeable difference in abundance between the two sediment types (Figure 7; Table S4, Appendix F).

Exploring treatment effects, VadinHA17_877549, Burkholderiaceae_A_592522, and Methylophilaceae had highest overall abundances in the high spiked treatment and Coleofasciculaceae_23353, Nostocaceae_28012, and Pseudanabaenaceae had lowest abundances in the high spiked treatment, regardless of sediment origin. Prolixibacteraceae, Geobacteraceae, and Rhodanobacteraceae_613062 had highest abundances in the high spiked treatment for the GLE samples but showed an opposite trend in the BIT samples. Chitinophagaceae_966727 had highest abundance in the high spiked treatment for BIT but showed no clear trend for GLE. Pseudopelobacteraceae had highest abundance in the high spiked treatment for GLE but showed no clear trend for BIT. Rhodocyclaceae had highest overall abundance in the GLE controls and BIT low spike samples (Figure 7; Table S4, Appendix F).

Looking at temporal dynamics, the overall trends were consistent across each treatment group for the BIT sediment samples, but not for the GLE sediment samples. For the BIT samples, Prolixibacteraceae, VadinHA17_877549, Coleofasciculaceae_23353, and Geobacteraceae all decreased in abundance over the duration of the experiment while Chitinophagaceae_966727 and Rhodanobacteraceae_613062 increased in abundance. Methylophilaceae and Burkholderiaceae_A_592522 began increasing then decreased at the end of the experimental period. Similarly, Pseudopelobacteraceae and Rhodocyclaceae began increasing before decreasing at the mid‐point of the experiment. Two families, Nostocaceae_28012 and Pseudanabaenaceae, did not exhibit any strong temporal trends. For the GLE samples, VadinHA17_877549, Chitinophagaceae_966727, and Rhodocyclaceae had identical trends as the BIT samples. In the controls, Prolixibacteraceae and Pseudopelobacteraceae had identical trends to BIT, Nostocaceae_28012 and Burkholderiaceae_A_592522 increased over time, Coleofasciculaceae_23353 and Geobacteraceae increased before decreasing mid‐way, Pseudanabaenaceae increased before decreasing at the end, Methylophilaceae abundances fluctuated over time, and Rhodanobacteraceae_613062 showed no strong temporal trend. In the low spike samples, Methylophilaceae had an identical trend to BIT, Nostocaceae_28012, Pseudanabaenaceae, and Burkholderiaceae_A_592522 increased over time, Pseudopelobacteraceae decreased over time, Coleofasciculaceae_23353 increased then decreased mid‐way, Prolixibacteraceae increased at the first spike then deceased, and Geobacteraceae and Rhodanobacteraceae_613062 showed no strong temporal trend. In the high spike samples, Nostocaceae_28012, Pseudanabaenaceae, and Pseudopelobacteraceae had identical trends to BIT, Burkholderiaceae_A_592522 increased over time, Prolixibacteraceae and Geobacteraceae increased before decreasing mid‐way, Coleofasciculaceae_23353 spiked in abundance at the end of the experiment, Methylophilaceae fluctuated, and Rhodanobacteraceae_613062 showed no strong temporal trend (Figure 7; Table S4, Appendix F).

BEST analysis (Table 3) was used to assess the correlation between chemical or environmental variables and microbial structure, to evaluate their influence on shaping microbial assemblage structures. The microbial assemblages in each sample were analysed based on identification of taxa at ASV level and compared against eight variables: copper, lead, nickel, zinc, arsenic, cadmium, chromium, and pH (Table S3, Appendix C). For both sediments, the BEST analysis determined that the best‐fit model for a single environmental variable was pH, and the best‐fit model for two variables was pH and copper. Both sediments showed a similar trend in which there was a larger increase in the correlation value between one and two variables, followed by steady or unchanging correlations from two through eight variables. Although they follow the same trend, the correlation values for BIT were higher than that of GLE.

**TABLE 3:** BEST analysis results for BIT and GLE samples, determined from 16S DNA, and eight environmental variables.

No. variables	Correlation	Variables
BIT		
1	0.672	pH
2	0.681	Cu, pH
3	0.681	Cu, Cr, pH
4	0.681	Cu, Pb, Cr, pH
5	0.681	Cu, Pb, Zn, Cr, pH
6	0.681	Cu, Pb, Zn, As, Cr, pH
7	0.681	Cu, Pb, Zn, As, Cd, Cr, pH
8	0.681	Cu, Pb, Ni, Zn, As, Cd, Cr, pH
GLE		
1	0.430	pH
2	0.432	Cu, pH
3	0.432	Cu, As, pH
4	0.432	Cu, As, Cd, pH
5	0.432	Cu, Ni, As, Cd, pH
6	0.432	Cu, Zn, As, Cd, Cr, pH
7	0.431	Cu, Ni, Zn, As, Cd, Cr, pH
8	0.356	Cu, Pb, Ni, Zn, As, Cd, Cr, pH

## 4 Discussion

Urban streams and wetlands are often contaminated with a range of heavy metals (Flynn et al. 2025; Sharley et al. 2017). Cu binds strongly to particulate matter and accumulates in sediments (ANZG 2018; Rader et al. 2019), so is expected to displace heavy metals already present in the environment and bound to sediments, releasing them into the surrounding water (Simpson et al. 2000; Wojtkowska 2013). Therefore, a Cu contamination event is predicted to cause a complex set of chemical reactions, increasing the bioavailability of other metals present in the system and raising the toxicity of the water. We anticipated that the microbial assemblages would be affected by and respond to the combined physicochemical consequences and increase in overall toxicity as a result of the addition of CuSO_4_, as well as the increasing Cu concentration itself.

Various bacteria have evolved systems to tolerate exposure to contaminants through mechanisms such as efflux, sequestration, and redox (Mathivanan et al. 2021). In our study, the BIT samples, which had high abundances of Cyanobacteria initially, maintained higher abundances in the control samples with equal abundances in the two spiked treatments. Conversely, the GLE samples, which had less Cyanobacteria present initially, showed clearer separation between each of the treatments, with abundances reducing incrementally as Cu concentrations increased. Cyanobacteria are known to be sensitive to heavy metals, Cu in particular (Tchounwou et al. 2012), including Coleofasciculaceae (Feng et al. 2024) and Nostocaceae (Cepoi et al. 2022), which were two of the thirteen families that accounted for the most variation in bacterial assemblage structure. In low concentrations Nostocaceae have been observed to bioaccumulate metals including Cd, Zn, Ni, and Cu, with multi‐metal systems in particular aiding in increased resistance (Cepoi et al. 2022; El‐Enany and Issa 2000). Nostocaceae were detected in the DGV treatments towards the end of the experiment; however, only in the GLE samples. Coleofasciculaceae had a clear decline in abundance with increasing Cu concentration, particularly in the BIT samples. Similarly, Patescibacteria also exhibited the same responses as Cyanobacteria for both the BIT and GLE samples. The sensitivity to contamination observed for Patescibacteria in this study aligns with the results found in other studies (Tian et al. 2020). Myxococcota only contributed significantly to variation between treatments in the BIT samples, where it showed equal tolerance for the control and DGV treatments, and a sensitivity to the HGV treatment. Some previous studies also found a negative correlation between Myxococcota and metal contamination (Zuo et al. 2023), while others identified Myxococcota to be persistent in contaminated environments (Hao et al. 2021).

While Cyanobacteria, Patescibacteria, and Myxococcota displayed sensitivity to metal contamination, Proteobacteria, Desulfobacterota, and Bacteroidota displayed a tolerance for the contaminated treatments. Proteobacteria, a phylum known for its extensive metal tolerance (Chen et al. 2018; Guo et al. 2018), showed greatest abundance in the Cu spiked BIT treatments and equal greatest abundance in the control and HGV GLE treatments. The high relative abundance of Proteobacteria in the GLE control could be attributed to fact that the TU quotient for the GLE control samples was almost identical to that of the BIT DGV treatment. The GLE DGV sample had a slightly lower TU compared to the other treatments, which accounts for the reduced Proteobacteria in those samples. Four Proteobacteria families contributed significantly to assemblage variation: Burkholderiaceae, Rhodocyclaceae, Methylophilaceae, and Rhodanobacteraceae. The Burkholderia genus in particular has a reputation for being ubiquitous and having a high tolerance for metals (Lan et al. 2023), and in this study showed a consistent increase in abundance with increasing contamination. Methylophilaceae and Rhodocyclaceae both showed an increasing abundance that was positively correlated with high Zn TU, similar to what was seen in (Ni et al. 2016). Rhodanobacteraceae was only present on day 28 in the spiked GLE treatments and was present in the BIT samples from day 11 onwards, with higher concentrations in the spiked treatments until day 56 where abundance was highest in the control and lowest in the HGV. These inconsistent trends across treatment groups suggest that their abundance may have been influenced more significantly by the time spent in the microcosm environment rather than the concentration of contaminants to which they were exposed. Desulfobacterota have been associated with highly metal contaminated environments (Hao et al. 2021) and biodegrade heavy metals from acid mine drainage (Yan et al. 2023). Desulfobacterota was also associated with high metal concentrations in this study, with higher abundances in the spiked treatments and an increasing abundance in HGV compared to DGV in the GLE samples. Bacteroidota had an equal increased abundance for both the spiked treatments in the BIT samples, while the GLE samples showed clear increase in abundance from the control to the DGV to the HGV treatment. Bacteroidota sequences have been found to contain Cu resistance genes (*cusC*) (Galisteo et al. 2024), and the phylum has also been previously associated with Cd contamination (Wu et al. 2017), substantiating their tolerance for metal contamination and preference for the spiked treatments. Three families contributed most to the variation seen: Prolixibacteraceae which was highly positively correlated with contaminant concentrations in the GLE samples, and Chitinophagaceae and VadinHA17_877549 which showed a slight positive correlation with contaminant concentrations but seemed to be correlated more with time. Prolixibacteraceae has been previously associated with acid mine drainage, which is known to contain high levels of heavy metals (Florentino et al. 2015; Oyetibo et al. 2021). Despite the slight increases in abundance seen with increasing contamination in this study, VadinHA17_877549 and Chitinophagaceae have both previously been found to be negatively impacted by metal contamination (Flynn et al. 2025; Hermans et al. 2017; Rogiers et al. 2021; Yang et al. 2024).

In our study, besides the addition of Cu to the treatments via the spikes (and consequent mobilisation of latent metals from the sediment), the water quality parameter that varied most throughout the course of the experiment was pH, which had a steady decline over the 8‐week experimental period. This increase in acidity was expected, resulting from the production of sulphuric acid via an exchange of ions from the CuSO4. Increasing acidity consequently increases the toxicity of metals, as more ions are present in solution (Wang et al. 2016). Taxa that showed more variation between time groups compared to treatment groups within each time point may be more influenced by physicochemical parameters, like pH, rather than metal contamination. Pseudanabaenaceae and Rhodanobacteraceae experienced a slight increase in abundance as pH reduced over time. While Rhodanobacteraceae has been found to be positively correlated with lower pH (Green et al. 2012; Lidor et al. 2019), Pseudanabaenaceae has been associated with neutral to higher pH (Kamran et al. 2020; Kees et al. 2022), contrasting with what was seen in this study. Contrarily, Geobacteraceae and Pseudopelobacteraceae experienced a slight decrease in abundance over time. Geobacteraceae is usually associated with neutral pH (Xu et al. 2020; Yuan et al. 2016) but has been seen in high pH conditions (Yuan et al. 2016), aligning with our results. Previous studies have not explored the relationship between pH and Pseudopelobacteraceae abundance, but the results from this study show a slight preference for neutral or alkaline conditions. Although metal concentrations increased over the course of the experiment, taxa that are either sensitive to or tolerant of metals are expected to show variation between treatment groups as well as over time.

Various studies have found that bacterial assemblages contrast significantly depending on the level and type of contamination to which they are exposed (Flynn et al. 2025; Wu et al. 2017). This concept was also demonstrated in our study, with the local assemblage phyla differing between the uncontaminated and contaminated sediments. Of the phyla that contributed most to this disparity, Cyanobacteria and Campylobacterota were more dominant at BIT, and Proteobacteria, Bacteroidota, Verrucomicrobiota, Patescibacteria, and Chloroflexota were more dominant at GLE on day 0 of the experiment. The differentiation between these two sediment assemblages was evident in their varied responses to the Cu contamination exposures both over a concentration and time gradient, as described above. Overall, at a phylum level the bacterial assemblages of the BIT samples proved to be a better indicator for the presence of contamination, while the assemblages from the GLE samples were better at differentiating between concentrations of contaminants. At a family level however, the community from BIT becomes more useful in differentiating between concentrations. This is slightly unexpected as it is assumed that the microbiome in the BIT samples will have a higher proportion of sensitive taxa, resulting from low background pollution, while the GLE samples will have a higher proportion of tolerant taxa, resulting from high levels of background pollution; also assuming that a higher proportion of sensitive taxa will result in a more sensitive indication of contamination. Although the BEST analysis results showed a higher correlation between the environmental variables and microbial composition for the BIT samples compared to the GLE samples, which does align with this assumption.

Of the important bacterial families identified in this study, seven have been previously identified as having relevant potential bioindicative ability through field surveys (Flynn et al. 2025). Coleofasciculaceae, Chitinophagaceae, Pseudopelobacteraceae, Burkholderiaceae, VadinHA17_877549, Rhodocyclaceae, and Methylophilaceae were found to exhibit the same responses to contamination (particularly zinc and copper) as we saw in this study, under comparable conditions (i.e., sediment type, pollution profile, contaminant concentration). Coleofasciculaceae, Pseudopelobacteraceae, VadinHA17_877549, and Methylophilaceae were negatively correlated with increasing contamination (primarily depleted, sensitive) while Chitinophagaceae, Burkholderiaceae, and Rhodocyclaceae were positively correlated with increasing contamination (primarily enriched, tolerant). While most of these families are taxonomically stable, the provisional database‐derived labels assigned to some of the taxa lead them to being more indicative of community dynamics rather than suggestions of taxa which could yet be added to a bacterial bioindicator index.

Overall, we found that the higher concentration exposure did result in a stronger microbial response, that responses are impacted by the presence of previous contamination, and that bacterial shifts develop over time with some taxa showing signs of stabilisation after 8 weeks of exposure. A significant factor expected to impact communities within microcosms, alongside impacts from the assessed chemical variables, is incubation effects from the microcosm environment itself. These effects, however, were accounted for in our analysis through the collection of control samples at every time point which enabled direct comparison between the treatments and controls at the same time point, allowing for assessment of chemical effects without interference from temporal changes. It is still important to note that the responses seen in this study are a result of combined metal toxicity and acidification from CuSO_4_ spiking, rather than a direct copper‐ion effect, in a controlled model environment. The complexities of a dynamic freshwater system are difficult to reproduce under laboratory conditions; therefore the results presented here although indicative may not directly reflect the dynamics of microbial responses in a natural system.

While our study found significant variation in microbial assemblage composition correlated with metal contamination as a result of a Cu pollution event, further research is needed to assess the impacts of different classes of contaminants (to determine if the responses seen in this study are general pollution responses or contaminant specific) and in a wider range of environments before the potential microbial bioindicators identified here can be confirmed as suitable for use in biomonitoring and ecological assessments. The use of sedimentary eDNA also restricts investigation to only what DNA has persisted in the environment at the time of sampling and limits analysis to including both living and dead biomass due to the uncertainty of interpreting DNA signals as direct proxies for living or active biomass. Additionally, the inclusion of functional gene analysis alongside microbial structural analysis would provide further support of the usefulness of these taxa as bioindicators of metal pollution through the identification of the genes responsible for tolerance and consequent understanding of how these microbial taxa withstand high metal concentrations. Functional gene analysis could also likely aid in understanding the potential implications of microbial functioning on the overall performance of CWs.

## 5 Conclusion

In this study we were able to describe the local microbial communities from an unpolluted and a polluted constructed wetland and assess the variation in their structural responses to a copper sulphate contamination event of increasing concentrations. Chemistry results showed that the addition of copper into the microcosms not only increased the concentration of copper but also displaced latent metals in the sediments and decreased microcosm pH, increasing the overall toxicity and bioavailability of multiple metals in the system. Significant variation was found between each time point as well as between treatments, across both sediments. With the addition of copper to the microcosms, the microbial communities in both sediments' spiked treatments shifted, becoming more similar as a result. Seven families from four phyla were determined to exhibit consistent responses to metal contamination, or its consequent effects on water chemistry. We found that the correlation factor between microbial community structure and environmental variables is affected by the background concentrations of contamination as well as the taxonomic level of the analysis. This study demonstrated the complex chemical reactions that occur during a copper contamination event in an urban stream or wetland and characterised the responses of sedimentary microbial communities to these changes.

## Author Contributions

**Anna S. Flynn:** conceptualization (lead), formal analysis (lead), investigation (lead), methodology (lead), writing – original draft (lead), writing – review and editing (equal). **A. Mark Osborn:** conceptualization (supporting), methodology (supporting), writing – review and editing (equal). **Vincent Pettigrove:** conceptualization (supporting), methodology (supporting), writing – review and editing (equal). **Jeff Shimeta:** conceptualization (supporting), methodology (supporting), writing – review and editing (equal). **Sara M. Long:** conceptualization (supporting), methodology (supporting), writing – review and editing (equal).

## Funding

This work was supported by Department of Education, Australian Government. Melbourne Water.

## Conflicts of Interest

The authors declare no conflicts of interest.

## Supporting information


**Figure S1:** Metal concentrations measured from BIT and GLE sediment samples at five time points (0, 2, 11, 28, 56) for three treatments (control, DGV, HGV). A grey dotted line represents the ANZG [12] default guideline value for sediment and a black dotted line represents the high guideline value. A red dotted line indicates when the initial spiking occurred.
**Table S1:** Detection limits and ANZG [12] 80% trigger values for water.
**Table S2:** Detection limits and ANZG [12] and default guideline value (DGV) & high guideline value (HGV) trigger values for sediment.
**Table S3:** pH measurements of both sediments (BIT, GLE) for each treatment (control, DGV, HGV) at each time point (Day 0, 2, 11, 28, 56).
**Figure S2:** nMDS ordination plot of all microcosm samples, determined from 16S DNA at phylum level. Samples are split by time (day 0, 2, 11, 28, 56) and sediment (BIT, GLE).
**Table S4:** SIMPER analysis results comparing families between treatments (control, DGV, HGV) at each time point (day 0, 2, 11, 28, 56). Average abundance (Av. Abun), contribution (Cont. %) and cumulative contribution (Cum. %) are shown for the top 5 contributing taxa. Taxa that could not be classified down to family are written as “Phylum; *lowest possible classification*; f__”.
**Equation S1:** Sediment moisture content equation.
**Equation S2:** Microcosm spiking volume equation.
**Equation S3:** Toxic Unit (TU) equation using the measured concentration of metal in the water samples and the ANZG [12] 80% trigger value adjusted for water hardness.

## Data Availability

The sequence data have been submitted to the NCBI Sequence Read Archive (SRA) database under accession number PRJNA1191220.
