# Supplementary material for: eDNA Analysis of Aquatic Microcosms Reveals the Effects of a Copper Pollution Event on Sedimentary Microbial Assemblages in Freshwater Wetlands
Source: Ecol Evol. 2026 Aug 11;16(8):e74180. doi: 10.1002/ece3.74180 (PMC13462677; doi:10.1002/ece3.74180)
Supplement: Supplementary file 1 [file ECE3-16-e74180-s001.zip › Appendices.docx]

**Appendices**

**Appendix A**

**Equation S1.** Sediment moisture content equation.

$\frac{Wet sediment weight \left( g \right)-Dry sediment weight (g)}{Wet sediment weight (g)} =Moisture content$

**Equation S2.** Microcosm spiking volume equation.

The weight of the empty beaker (g, E) was subtracted from the sediment filled beaker (g, F) and divided by 1000 to get the wet sediment weight (kg, w).

$$\frac{F-E}{1000} =w$$

The wet sediment weight (kg, w) was multiplied by one minus the sediment moisture content (M) to get the dry sediment weight (kg, d).

$w \times\left( 1 -M \right)=d$

The dry sediment weight (kg, d) was multiplied by the final desired Cu concentration (mg/kg, C) to get the amount of Cu needed to be added (mg, c) to achieve the final concentration.

$$d \times C =c$$

The amount of Cu needed to be added (mg, c) was divided by the concentration of the stock solution (mg/L, S) to get the spiking volume (v, L).

$$\frac{c}{S} =v$$

Finally, the spiking volume (v, L) was multiplied by 1000000 to get the final spiking volume (x, µL) in microlitres.

$$v \times1000000 =x$$

**Equation S3.** Toxic Unit (TU) equation using the measured concentration of metal in the water samples and the ANZG [12] 80% trigger value adjusted for water hardness.

$$\sum\frac{chemical concentration (mg/L)}{80\% trigger value (mg/L)}$$

**Appendix B**

**Table S1.** Detection limits and ANZG [12] 80% trigger values for water.

|  | **Metal (mg/L)** | | | | | | |
| --- | --- | --- | --- | --- | --- | --- | --- |
|  | **Arsenic** | **Cadmium** | **Chromium** | **Copper** | **Lead** | **Nickel** | **Zinc** |
| **Detection limit** | <0.001 | <0.0001 | <0.001 | <0.001 | <0.001 | <0.001 | <0.005 |
| **80% trigger** | 0.14 | 0.0008 | 0.04 | 0.0025 | 0.0094 | 0.017 | 0.031 |

**Table S2.** Detection limits and ANZG [12] and default guideline value (DGV) & high guideline value (HGV) trigger values for sediment.

|  | **Metal (mg/kg)** | | | | | | |
| --- | --- | --- | --- | --- | --- | --- | --- |
|  | **Arsenic** | **Cadmium** | **Chromium** | **Copper** | **Lead** | **Nickel** | **Zinc** |
| **Detection limit** | <5 | <1 | <2 | <5 | <5 | <2 | <5 |
| **DGV** | 20 | 2 | 80 | 65 | 50 | 21 | 200 |
| **HGV** | 70 | 25 | 370 | 270 | 220 | 52 | 410 |

**Appendix C**

**Table S3.** pH measurements of both sediments (BIT, GLE) for each treatment (control, DGV, HGV) at each time point (Day 0, 2, 11, 28, 56).

| **Sediment** | **Treatment** | **Day** | **pH** |
| --- | --- | --- | --- |
| BIT | Control | 0 | 7.00 |
|  | DGV |  | 7.03 |
|  | HGV |  | 6.98 |
|  | Control | 2 | 6.90 |
|  | DGV |  | 7.03 |
|  | HGV |  | 6.96 |
|  | Control | 11 | 5.15 |
|  | DGV |  | 6.14 |
|  | HGV |  | 5.96 |
|  | Control | 28 | 6.04 |
|  | DGV |  | 6.04 |
|  | HGV |  | 6.53 |
|  | Control | 56 | 5.19 |
|  | DGV |  | 4.95 |
|  | HGV |  | 5.14 |
| GLE | Control | 0 | 6.63 |
|  | DGV |  | 6.43 |
|  | HGV |  | 6.46 |
|  | Control | 2 | 6.89 |
|  | DGV |  | 6.72 |
|  | HGV |  | 6.49 |
|  | Control | 11 | 5.79 |
|  | DGV |  | 5.83 |
|  | HGV |  | 5.80 |
|  | Control | 28 | 6.05 |
|  | DGV |  | 5.86 |
|  | HGV |  | 5.56 |
|  | Control | 56 | 6.03 |
|  | DGV |  | 5.91 |
|  | HGV |  | 5.53 |

**Appendix D**

**Figure S1.** Metal concentrations measured from BIT and GLE sediment samples at five time points (0, 2, 11, 28, 56) for three treatments (control, DGV, HGV). A grey dotted line represents the ANZG [12] default guideline value for sediment and a black dotted line represents the high guideline value. A red dotted line indicates when the initial spiking occurred.

**Appendix E**

**Figure S2.** nMDS ordination plot of all microcosm samples, determined from 16S DNA at phylum level. Samples are split by time (day 0, 2, 11, 28, 56) and sediment (BIT, GLE).

**Appendix F**

**Table S4**. SIMPER analysis results comparing families between treatments (control, DGV, HGV) at each time point (day 0, 2, 11, 28. 56). Average abundance (Av. Abun), contribution (Cont. %) and cumulative contribution (Cum. %) are shown for the top 5 contributing taxa. Taxa that could not be classified down to family are written as “Phylum; *lowest possible classification*; f__”.

| **BIT** | | | | |
| --- | --- | --- | --- | --- |
| **Day 0** | | | | |
| **Control – DGV (Average dissimilarity = 10.64)** | | | | |
| **Taxa** | **Av. Abun (C)** | **Av. Abun (DGV)** | **Cont. %** | **Cum. %** |
| Cyanobacteria; f__Coleofasciculaceae_23353 | 0.19 | 0.16 | 15.77 | 15.77 |
| Desulfobacterota_G_459546; f__Geobacteraceae | 0.03 | 0.03 | 3.12 | 18.89 |
| Desulfobacterota_G_459546;f__Pseudopelobacteraceae | 0.02 | 0.02 | 2.54 | 21.42 |
| d__Bacteria;__;__;__;__ | 0.03 | 0.03 | 1.6 | 23.03 |
| Desulfobacterota_G_459546f__BM103 | 0.01 | 0.01 | 1.51 | 24.53 |
| **Control – HGV (Average dissimilarity = 10.25)** | | | | |
| **Taxa** | **Av. Abun (C)** | **Av. Abun (HGV)** | **Cont. %** | **Cum. %** |
| Cyanobacteria; f__Coleofasciculaceae_23353 | 0.19 | 0.15 | 16.1 | 16.1 |
| Bacteroidota;c__Bacteroidia;f__ | 0.03 | 0.02 | 2.09 | 18.19 |
| d__Bacteria;__;__;__;__ | 0.03 | 0.03 | 1.95 | 20.15 |
| Desulfobacterota_G_459546; f__BM103 | 0.01 | 0.02 | 1.59 | 21.74 |
| **DGV – HGV (Average dissimilarity = 9.55)** | | | | |
| **Taxa** | **Av. Abun (DGV)** | **Av. Abun (HGV)** | **Cont. %** | **Cum. %** |
| Cyanobacteria; f__Coleofasciculaceae_23353 | 0.16 | 0.15 | 8.03 | 8.03 |
| Desulfobacterota_G_459546; f__Geobacteraceae | 0.03 | 0.03 | 2.91 | 10.94 |
| Bacteroidota;c__Bacteroidia;f__ | 0.03 | 0.02 | 2.42 | 13.36 |
| Desulfobacterota_G_459546; f__Pseudopelobacteraceae | 0.02 | 0.02 | 1.95 | 15.31 |
| d__Bacteria;__;__;__;__ | 0.03 | 0.03 | 1.56 | 16.87 |
| **Day 2** | | | | |
| **Control – DGV (Average dissimilarity = 11.71)** | | | | |
| **Taxa** | **Av. Abun (C)** | **Av. Abun (DGV)** | **Cont. %** | **Cum. %** |
| Cyanobacteria; f__Coleofasciculaceae_23353 | 0.15 | 0.15 | 7.8 | 7.8 |
| Desulfobacterota_G_459546; f__Pseudopelobacteraceae | 0.03 | 0.04 | 5.78 | 13.58 |
| Desulfobacterota_G_459546; f__Geobacteraceae | 0.03 | 0.04 | 4.14 | 17.71 |
| Bacteroidota; f__VadinHA17_877549 | 0.04 | 0.03 | 2.59 | 20.3 |
| Desulfobacterota_I; f__Desulfatiglandaceae | 0 | 0.01 | 2.29 | 22.59 |
| **Control – HGV (Average dissimilarity = 10.84)** | | | | |
| **Taxa** | **Av. Abun (C)** | **Av. Abun (HGV)** | **Cont. %** | **Cum. %** |
| Cyanobacteria; f__Coleofasciculaceae_23353 | 0.15 | 0.16 | 6.04 | 6.04 |
| Desulfobacterota_G_459546; f__Pseudopelobacteraceae | 0.03 | 0.04 | 4 | 10.05 |
| Desulfobacterota_I; f__Desulfatiglandaceae | 0 | 0.01 | 2.21 | 12.25 |
| Desulfobacterota_G_459546; f__Geobacteraceae | 0.03 | 0.04 | 1.97 | 14.23 |
| Bacteroidota; f__VadinHA17_877549 | 0.04 | 0.03 | 1.89 | 16.11 |
| **DGV – HGV (Average dissimilarity = 9.93)** | | | | |
| **Taxa** | **Av. Abun (DGV)** | **Av. Abun (HGV)** | **Cont. %** | **Cum. %** |
| Cyanobacteria; f__Coleofasciculaceae_23353 | 0.15 | 0.16 | 10.65 | 10.65 |
| Desulfobacterota_G_459546;f__Pseudopelobacteraceae | 0.04 | 0.04 | 4.42 | 15.07 |
| Desulfobacterota_G_459546; f__Geobacteraceae | 0.04 | 0.04 | 2.88 | 17.95 |
| Proteobacteria; f__Rhodocyclaceae | 0.02 | 0.01 | 2.26 | 20.21 |
| Bacteroidota; f__VadinHA17_877549 | 0.03 | 0.03 | 1.35 | 21.56 |
| **Day 11** | | | | |
| **Control – DGV (Average dissimilarity = 17.06)** | | | | |
| **Taxa** | **Av. Abun (C)** | **Av. Abun (DGV)** | **Cont. %** | **Cum. %** |
| Campylobacterota; o__Campylobacterales;f__ | 0.06 | 0.03 | 10.62 | 10.62 |
| Proteobacteria; f__Burkholderiaceae_A_592522 | 0.01 | 0.04 | 6.92 | 17.54 |
| Cyanobacteria; f__Coleofasciculaceae_23353 | 0.15 | 0.14 | 6.1 | 23.64 |
| Proteobacteria;f__Rhodocyclaceae | 0.03 | 0.04 | 3.3 | 26.95 |
| Proteobacteria; f__Rhodanobacteraceae_613062 | 0.01 | 0 | 2.09 | 29.04 |
| **Control – HGV (Average dissimilarity = 21.93)** | | | | |
| **Taxa** | **Av. Abun (C)** | **Av. Abun (HGV)** | **Cont. %** | **Cum. %** |
| Proteobacteria; f__Burkholderiaceae_A_592522 | 0.01 | 0.06 | 11.56 | 11.56 |
| Campylobacterota; o__Campylobacterales;f__ | 0.06 | 0.03 | 7.36 | 18.92 |
| Cyanobacteria;f__Coleofasciculaceae_23353 | 0.15 | 0.12 | 6.78 | 25.69 |
| Proteobacteria; f__Rhodanobacteraceae_613062 | 0.01 | 0.02 | 3.41 | 29.1 |
| Proteobacteria; f__Rhodocyclaceae | 0.03 | 0.02 | 3.38 | 32.49 |
| **DGV – HGV (Average dissimilarity = 17.47)** | | | | |
| **Taxa** | **Av. Abun (DGV)** | **Av. Abun (HGV)** | **Cont. %** | **Cum. %** |
| Proteobacteria; f__Burkholderiaceae_A_592522 | 0.04 | 0.06 | 7.74 | 7.74 |
| Cyanobacteria; f__Coleofasciculaceae_23353 | 0.14 | 0.12 | 6.36 | 14.1 |
| Proteobacteria; f__Rhodanobacteraceae_613062 | 0 | 0.02 | 5.53 | 19.63 |
| Desulfobacterota_G_459546; f__Pseudopelobacteraceae | 0.04 | 0.03 | 4.93 | 24.56 |
| Proteobacteria; f__Rhodocyclaceae | 0.04 | 0.02 | 4 | 28.56 |
| **Day 28** | | | | |
| **Control – DGV (Average dissimilarity = 36.54)** | | | | |
| **Taxa** | **Av. Abun (C)** | **Av. Abun (DGV)** | **Cont. %** | **Cum. %** |
| Cyanobacteria; f__Coleofasciculaceae_23353 | 0.14 | 0.1 | 8.68 | 8.68 |
| Proteobacteria; f__Burkholderiaceae_A_574758 | 0.08 | 0.04 | 8.02 | 16.7 |
| Myxococcota_A_473307; f__Myxococcaceae | 0.03 | 0.02 | 4.64 | 21.34 |
| Proteobacteria;f__Burkholderiaceae_A_592522 | 0.07 | 0.09 | 4.52 | 25.86 |
| Proteobacteria; f__Gallionellaceae | 0.01 | 0.04 | 3.37 | 29.23 |
| **Control – HGV (Average dissimilarity = 44.07)** | | | | |
| **Taxa** | **Av. Abun (C)** | **Av. Abun (HGV)** | **Cont. %** | **Cum. %** |
| Cyanobacteria; f__Coleofasciculaceae_23353 | 0.14 | 0.04 | 12.04 | 12.04 |
| Proteobacteria; f__Burkholderiaceae_A_574758 | 0.08 | 0.02 | 7.92 | 19.96 |
| Bacteroidota; f__Chitinophagaceae_966727 | 0.01 | 0.07 | 7.49 | 27.44 |
| Myxococcota_A_473307; f__Myxococcaceae | 0.03 | 0.02 | 3.97 | 31.42 |
| Proteobacteria; f__Burkholderiaceae_A_592522 | 0.07 | 0.08 | 3.65 | 35.07 |
| **DGV – HGV (Average dissimilarity = 37.13)** | | | | |
| **Taxa** | **Av. Abun (DGV)** | **Av. Abun (HGV)** | **Cont. %** | **Cum. %** |
| Cyanobacteria; f__Coleofasciculaceae_23353 | 0.1 | 0.04 | 8.94 | 8.94 |
| Bacteroidota; f__Chitinophagaceae_966727 | 0.01 | 0.07 | 8.37 | 17.31 |
| Proteobacteria; f__Gallionellaceae | 0.04 | 0.02 | 3.65 | 20.96 |
| Proteobacteria; f__Burkholderiaceae_A_574758 | 0.04 | 0.02 | 3.29 | 24.26 |
| Proteobacteria; f__Methylophilaceae | 0.01 | 0.03 | 3.02 | 27.27 |
| **Day 56** | | | | |
| **Control – DGV (Average dissimilarity = 45.18)** | | | | |
| **Taxa** | **Av. Abun (C)** | **Av. Abun (DGV)** | **Cont. %** | **Cum. %** |
| Proteobacteria; f__Xanthomonadaceae_616009 | 0.11 | 0 | 12.27 | 12.27 |
| Cyanobacteria;c__Cyanobacteriia;f__ | 0.08 | 0 | 8.98 | 21.24 |
| Campylobacterota;o__Campylobacterales;f__ | 0.05 | 0.07 | 5.15 | 26.39 |
| Proteobacteria; f__Beijerinckiaceae | 0.05 | 0.01 | 4.53 | 30.93 |
| Proteobacteria; f__Sulfuricellaceae_A | 0.02 | 0.06 | 4.37 | 35.29 |
| **Control – HGV (Average dissimilarity = 49.04)** | | | | |
| **Taxa** | **Av. Abun (C)** | **Av. Abun (HGV)** | **Cont. %** | **Cum. %** |
| Proteobacteria; f__Xanthomonadaceae_616009 | 0.11 | 0 | 11.32 | 11.32 |
| Cyanobacteria;c__Cyanobacteriia;f__ | 0.08 | 0 | 8.29 | 19.61 |
| Proteobacteria; f__Sulfuricellaceae_A | 0.02 | 0.09 | 7.4 | 27.01 |
| Cyanobacteria; f__Leptolyngbyaceae | 0 | 0.05 | 4.95 | 31.96 |
| Proteobacteria; f__Beijerinckiaceae | 0.05 | 0 | 4.53 | 36.49 |
| **DGV – HGV (Average dissimilarity = 30.79)** | | | | |
| **Taxa** | **Av. Abun (DGV)** | **Av. Abun (HGV)** | **Cont. %** | **Cum. %** |
| Campylobacterota; o__Campylobacterales;f__ | 0.07 | 0.06 | 6.16 | 6.16 |
| Proteobacteria; f__Sulfuricellaceae_A | 0.06 | 0.09 | 5.71 | 11.88 |
| Cyanobacteria; f__Leptolyngbyaceae | 0.04 | 0.05 | 5.04 | 16.91 |
| Bacteroidota; f__Chitinophagaceae_966727 | 0.03 | 0.05 | 4.88 | 21.8 |
| Proteobacteria;c__Gammaproteobacteria;f__; | 0.05 | 0.05 | 4.73 | 26.53 |
| **GLE** | | | | |
| **Day 0** | | | | |
| **Control – DGV (Average dissimilarity = 15.50)** | | | | |
| **Taxa** | **Av. Abun (C)** | **Av. Abun (DGV)** | **Cont. %** | **Cum. %** |
| Bacteroidota; f__Prolixibacteraceae | 0.11 | 0.08 | 9.77 | 9.77 |
| Desulfobacterota_G_459546; f__Pseudopelobacteraceae | 0.04 | 0.05 | 3.18 | 12.96 |
| Proteobacteria; f__Methylophilaceae | 0.04 | 0.03 | 2.82 | 15.77 |
| Proteobacteria; f__Methylomonadaceae | 0.03 | 0.03 | 2.48 | 18.25 |
| Verrucomicrobiota; f__UBA8199 | 0.01 | 0.01 | 2.25 | 20.5 |
| **Control – HGV (Average dissimilarity = 19.19)** | | | | |
| **Taxa** | **Av. Abun (C)** | **Av. Abun (HGV)** | **Cont. %** | **Cum. %** |
| Bacteroidota; f__Prolixibacteraceae | 0.11 | 0.08 | 8.24 | 8.24 |
| Proteobacteria; f__Methylophilaceae | 0.04 | 0.05 | 5.1 | 13.33 |
| Proteobacteria; f__Rhodocyclaceae | 0.03 | 0.03 | 2.98 | 16.32 |
| Desulfobacterota_G_459546; f__Pseudopelobacteraceae | 0.04 | 0.04 | 2.38 | 18.7 |
| Bacteroidota;f__VadinHA17_877549 | 0.05 | 0.05 | 1.61 | 20.3 |
| **DGV – HGV (Average dissimilarity = 18.46)** | | | | |
| **Taxa** | **Av. Abun (DGV)** | **Av. Abun (HGV)** | **Cont. %** | **Cum. %** |
| Bacteroidota;f__Prolixibacteraceae | 0.08 | 0.08 | 5.32 | 5.32 |
| Proteobacteria;f__Methylophilaceae | 0.03 | 0.05 | 5.02 | 10.34 |
| Proteobacteria; f__Rhodocyclaceae | 0.03 | 0.03 | 3.44 | 13.77 |
| Proteobacteria; f__Methylomonadaceae | 0.03 | 0.03 | 2.28 | 16.05 |
| Desulfobacterota_G_459546; f__Pseudopelobacteraceae | 0.05 | 0.04 | 1.98 | 18.03 |
| **Day 2** | | | | |
| **Control – DGV (Average dissimilarity = 13.61)** | | | | |
| **Taxa** | **Av. Abun (C)** | **Av. Abun (DGV)** | **Cont. %** | **Cum. %** |
| Bacteroidota; f__Prolixibacteraceae | 0.07 | 0.11 | 13.96 | 13.96 |
| Desulfobacterota_G_459546; f__Pseudopelobacteraceae | 0.04 | 0.04 | 2.75 | 16.71 |
| Desulfobacterota_G_459546;f__Geobacteraceae | 0.02 | 0.02 | 2.57 | 19.28 |
| d__Bacteria;__;__;__;__ | 0.05 | 0.04 | 2.4 | 21.68 |
| Proteobacteria; f__Rhodocyclaceae | 0.03 | 0.04 | 2.11 | 23.78 |
| **Control – HGV (Average dissimilarity = 14.72)** | | | | |
| **Taxa** | **Av. Abun (C)** | **Av. Abun (HGV)** | **Cont. %** | **Cum. %** |
| Bacteroidota;f__Prolixibacteraceae | 0.07 | 0.1 | 10.49 | 10.49 |
| Desulfobacterota_G_459546;f__Pseudopelobacteraceae | 0.04 | 0.05 | 5.49 | 15.98 |
| d__Bacteria;__;__;__;__ | 0.05 | 0.04 | 3.53 | 19.51 |
| Proteobacteria; f__Rhodocyclaceae | 0.03 | 0.04 | 3.24 | 22.75 |
| Desulfobacterota_G_459546; f__Geobacteraceae | 0.02 | 0.03 | 2.73 | 25.48 |
| **DGV – HGV (Average dissimilarity = 11.25)** | | | | |
| **Taxa** | **Av. Abun (DGV)** | **Av. Abun (HGV)** | **Cont. %** | **Cum. %** |
| Bacteroidota; f__Prolixibacteraceae | 0.11 | 0.1 | 5.76 | 5.76 |
| Desulfobacterota_G_459546; f__Pseudopelobacteraceae | 0.04 | 0.05 | 4.33 | 10.09 |
| Verrucomicrobiota; f__UBA8199 | 0.02 | 0.02 | 2.97 | 13.06 |
| Verrucomicrobiota; f__UBA11358 | 0.01 | 0.02 | 2.72 | 15.78 |
| Verrucomicrobiota; o__Pedosphaerales;f__ | 0.02 | 0.02 | 2.27 | 18.05 |
| **Day 11** | | | | |
| **Control – DGV (Average dissimilarity = 14.72)** | | | | |
| **Taxa** | **Av. Abun (C)** | **Av. Abun (DGV)** | **Cont. %** | **Cum. %** |
| Cyanobacteria; f__Coleofasciculaceae_23353 | 0.05 | 0.05 | 8.19 | 8.19 |
| Proteobacteria; f__Methylomonadaceae | 0.05 | 0.04 | 3.39 | 11.58 |
| Proteobacteria; f__Methylophilaceae | 0.05 | 0.04 | 3.26 | 14.84 |
| Proteobacteria; f__Rhodocyclaceae | 0.06 | 0.06 | 3.15 | 17.99 |
| Bacteroidota; f__Prolixibacteraceae | 0.07 | 0.07 | 3.02 | 21.01 |
| **Control – HGV (Average dissimilarity = 22.22)** | | | | |
| **Taxa** | **Av. Abun (C)** | **Av. Abun (HGV)** | **Cont. %** | **Cum. %** |
| Bacteroidota; f__Prolixibacteraceae | 0.07 | 0.11 | 10.23 | 10.23 |
| Cyanobacteria; f__Coleofasciculaceae_23353 | 0.05 | 0.01 | 8.61 | 18.84 |
| Proteobacteria; f__Rhodocyclaceae | 0.06 | 0.05 | 4.19 | 23.03 |
| Proteobacteria; f__Methylophilaceae | 0.05 | 0.05 | 2.74 | 25.77 |
| Proteobacteria; f__Gallionellaceae | 0.03 | 0.02 | 2.6 | 28.36 |
| **DGV – HGV (Average dissimilarity = 20.29)** | | | | |
| **Taxa** | **Av. Abun (DGV)** | **Av. Abun (HGV)** | **Cont. %** | **Cum. %** |
| Bacteroidota; f__Prolixibacteraceae | 0.07 | 0.11 | 10.7 | 10.7 |
| Cyanobacteria; f__Coleofasciculaceae_23353 | 0.05 | 0.01 | 9.1 | 19.79 |
| Proteobacteria; f__Rhodocyclaceae | 0.06 | 0.05 | 2.99 | 22.79 |
| Bacteroidota; f__VadinHA17_877549 | 0.03 | 0.03 | 2.58 | 25.36 |
| Proteobacteria; f__Methylophilaceae | 0.04 | 0.05 | 2.56 | 27.93 |
| **Day 28** | | | | |
| **Control – DGV (Average dissimilarity = 26.86)** | | | | |
| **Taxa** | **Av. Abun (C)** | **Av. Abun (DGV)** | **Cont. %** | **Cum. %** |
| Cyanobacteria; f__Pseudanabaenaceae | 0.04 | 0.01 | 6.85 | 6.85 |
| Cyanobacteria; f__Coleofasciculaceae_23353 | 0.04 | 0.02 | 5.71 | 12.56 |
| Cyanobacteria; f__Nostocaceae_28012 | 0.02 | 0.01 | 3.92 | 16.48 |
| Bacteroidota; f__Prolixibacteraceae | 0.04 | 0.05 | 3.87 | 20.35 |
| Proteobacteria; f__Rhodocyclaceae | 0.05 | 0.03 | 3.81 | 24.16 |
| **Control – HGV (Average dissimilarity = 30.34)** | | | | |
| **Taxa** | **Av. Abun (C)** | **Av. Abun (HGV)** | **Cont. %** | **Cum. %** |
| Cyanobacteria;f__Coleofasciculaceae_23353 | 0.04 | 0 | 6.84 | 6.84 |
| Cyanobacteria; f__Pseudanabaenaceae | 0.04 | 0 | 6.55 | 13.39 |
| Bacteroidota;c__Bacteroidia;o__Bacteroidales;f__Prolixibacteraceae | 0.04 | 0.07 | 6.25 | 19.65 |
| Cyanobacteria; f__Nostocaceae_28012 | 0.02 | 0 | 3.44 | 23.08 |
| Proteobacteria; f__Rhodocyclaceae | 0.05 | 0.04 | 3.21 | 26.29 |
| **DGV – HGV (Average dissimilarity = 23.40)** | | | | |
| **Taxa** | **Av. Abun (DGV)** | **Av. Abun (HGV)** | **Cont. %** | **Cum. %** |
| Bacteroidota; f__Prolixibacteraceae | 0.05 | 0.07 | 4.84 | 4.84 |
| Patescibacteria; f__UBA5272 | 0.02 | 0 | 3.66 | 8.5 |
| Proteobacteria; f__Burkholderiaceae_A_592522 | 0.02 | 0.03 | 3.6 | 12.1 |
| Cyanobacteria; f__Coleofasciculaceae_23353 | 0.02 | 0 | 3.42 | 15.51 |
| Proteobacteria; f__Thiobacillaceae | 0.03 | 0.04 | 3.37 | 18.89 |
| **Day 56** | | | | |
| **Control – DGV (Average dissimilarity = 26.44)** | | | | |
| **Taxa** | **Av. Abun (C)** | **Av. Abun (DGV)** | **Cont. %** | **Cum. %** |
| Cyanobacteria; f__Nostocaceae_28012 | 0.05 | 0.05 | 8.42 | 8.42 |
| Cyanobacteria; f__Pseudanabaenaceae | 0 | 0.04 | 6.51 | 14.92 |
| Proteobacteria; f__Rhodocyclaceae | 0.05 | 0.03 | 5.2 | 20.12 |
| Cyanobacteria; f__Coleofasciculaceae_23353 | 0.01 | 0.03 | 4.89 | 25.02 |
| Bacteroidota; f__Cyclobacteriaceae_900466 | 0.01 | 0.03 | 4.39 | 29.41 |
| **Control – HGV (Average dissimilarity = 35.74)** | | | | |
| **Taxa** | **Av. Abun (C)** | **Av. Abun (HGV)** | **Cont. %** | **Cum. %** |
| Cyanobacteria;f__Nostocaceae_28012 | 0.05 | 0 | 7.44 | 7.44 |
| Bacteroidota; f__Cyclobacteriaceae_900466 | 0.01 | 0.06 | 6.55 | 13.99 |
| Proteobacteria; f__Thiobacillaceae | 0.03 | 0.06 | 4.63 | 18.61 |
| Bacteroidota; f__Prolixibacteraceae | 0.03 | 0.06 | 3.79 | 22.41 |
| Proteobacteria; f__Burkholderiaceae_A_592522 | 0.02 | 0.04 | 3.79 | 26.2 |
| **DGV – HGV (Average dissimilarity = 34.88)** | | | | |
| **Taxa** | **Av. Abun (DGV)** | **Av. Abun (HGV)** | **Cont. %** | **Cum. %** |
| Cyanobacteria;f__Nostocaceae_28012 | 0.05 | 0 | 7.33 | 7.33 |
| Bacteroidota; f__Cyclobacteriaceae_900466 | 0.03 | 0.06 | 5.3 | 12.62 |
| Cyanobacteria; f__Pseudanabaenaceae | 0.04 | 0 | 5.06 | 17.69 |
| Cyanobacteria;f__Coleofasciculaceae_23353 | 0.03 | 0.03 | 4.17 | 21.86 |
| Proteobacteria; f__Burkholderiaceae_A_592522 | 0.02 | 0.04 | 3.93 | 25.79 |
